# Long-Term Persistence with Injectable Therapy in Relapsing-Remitting Multiple Sclerosis: An 18-Year Observational Cohort Study

**DOI:** 10.1371/journal.pone.0123824

**Published:** 2015-04-13

**Authors:** Simon Zhornitsky, Jamie Greenfield, Marcus W. Koch, Scott B. Patten, Colleen Harris, Winona Wall, Katayoun Alikhani, Jodie Burton, Kevin Busche, Fiona Costello, Jeptha W. Davenport, Scott E. Jarvis, Dina Lavarato, Helene Parpal, David G. Patry, Michael Yeung, Luanne M. Metz

**Affiliations:** 1 Department of Clinical Neurosciences, Faculty of Medicine, University of Calgary; Calgary, Canada; 2 Department of Psychiatry, Faculty of Medicine, University of Calgary, Calgary, Canada; 3 Hotchkiss Brain Institute, Faculty of Medicine, University of Calgary, Calgary, Canada; Friedrich-Alexander University Erlangen, GERMANY

## Abstract

Disease modifying therapies (DMTs) reduce the frequency of relapses and accumulation of disability in multiple sclerosis (MS). Long-term persistence with treatment is important to optimize treatment benefit. This long-term, cohort study was conducted at the Calgary MS Clinic. All consenting adults with relapsing-remitting MS who started either glatiramer acetate (GA) or interferon-β 1a/1b (IFN-β) between January 1^st^, 1996 and July 1^st^, 2011 were included. Follow-up continued to February 1^st^, 2014. Time-to-discontinuation of the initial and subsequently-prescribed DMTs (switches) was analysed using Kaplan-Meier survival analyses. Group differences were compared using log-rank tests and multivariable Cox regression models. Analysis included 1471 participants; 906 were initially prescribed GA and 565 were initially prescribed IFN-β. Follow-up information was available for 87%; 29 (2%) were lost to follow-up and 160 (11%) moved from Southern Alberta while still using DMT. Median time-to-discontinuation of all injectable DMTs was 11.1 years. Participants with greater disability at treatment initiation, those who started treatment before age 30, and those who started between 2006 and 2011 were more likely to discontinue use of all injectable DMTs. Median time-to-discontinuation of the initial DMT was 8.6 years. Those initially prescribed GA remained on treatment longer. Of 610 participants who discontinued injectable DMT, 331 (54%) started an oral DMT, or a second-line DMT, or resumed injectable DMT after 90 days. Persistence with injectable DMTs was high in this long-term population-based study. Most participants who discontinued injectable DMT did not remain untreated. Further research is required to understand treatment outcomes and outcomes after stopping DMT.

## Introduction

Multiple sclerosis (MS) is a chronic, progressively disabling disease of the central nervous system that evolves throughout adulthood. Without treatment most people with MS develop secondary progressive MS (SPMS) [1]; it is unclear if current therapy prevents this. Two types of injectable disease modifying therapy (DMT), glatiramer acetate (GA) and interferon-β1a and 1b (IFN-β), reduce relapse rates and delay the accumulation of neurological injury in RRMS [2–5]. However, DMTs are meant to be long-term treatments, and clinical trials do not represent long-term management in the everyday clinical setting. Adherence, which includes consistently taking prescribed doses and remaining on treatment over the long-term (persistence) is necessary to optimize benefits but patients with MS have various motivations to miss doses and discontinue treatment. Long-term studies are needed to examine treatment outcomes but they are dependent on adherence to DMTs [6].

Persistence is considered one of the most important measures of drug utilization [6]. Factors that can improve persistence include patient education and support, consistency of the care provider, confidence in the physician, and shared decision making regarding treatment selection whereas depression, lack of hope, and cognitive impairment reduce adherence [7,8]. Additional MS-specific factors working against persistence are increased disability, perceived lack of efficacy, and adverse drug effects [9]. Studies of persistence with GA and IFN-β in MS show widely varying results. Short-term persistence with the initial DMT ranges from a high rate of 86% after 3 years [10] to a low rate of 43% after 1 year [11]. Variability in persistence may be attributed to differences in population characteristics, health care environments, class of drug evaluated, and study method.

In this long-term, cohort study we examined persistence with the use of all injectable DMTs and the initially prescribed injectable DMT (GA or IFN-β) in adults with RRMS. We also determined which factors impacted persistence and whether our patients remained untreated after stopping injectable treatment.

## Methods

This study was conducted at the Calgary MS Clinic between January 1^st^, 1996 and February 1st, 2014. The clinic provides population-based multidisciplinary care to patients with MS or suspected MS who live in Calgary and Southern Alberta. Over 95% of the cost of DMT for active RRMS has been paid by the provincial government since December 1998. First line injectable treatment options during this period included: subcutaneous IFN-β-1b (approved in Canada in July, 1995), subcutaneous GA (approved September, 1997), subcutaneous IFN-β-1a (approved February, 1998), and intramuscular IFN-β-1a (approved April, 1998). Patients receive individual education and written information from clinic physicians and nurses before treatment selection. Use of a checklist supports preparation for treatment initiation. Treatment selection explicitly includes consideration of patient perspectives. Patients are then taught injection technique by pharmaceutical industry-funded home health nurses. Patients are reviewed at least yearly by a neurologist and are encouraged to contact clinic nurses or industry-sponsored telephone support nurses if they experience a change in their symptoms or difficulty managing DMT. Reassessment occurs as needed between annual visits where response, tolerance and adherence are reviewed.

Government funding supplements MS clinic staffing (nursing and clerical) to enhance safety and patient adherence. Clinic processes assure collection of demographic (date of birth, gender) and clinical (disease onset date, Expanded Disability Status Scale [EDSS] score, relapse history) information at all visits. Provincial insurance requirements assure annual assessment. Data has been collected prospectively at treatment initiation and is updated annually. Date of treatment initiation is reported to the clinic by the nurses who provide injection training. Stop reasons are determined by discussion between patients and clinic nurses around the time of discontinuation and confirmed by chart review. Attempts to contact patients who seem to be lost to follow-up include letters to the patient’s family physician and to the patient asking them to contact the clinic. These processes ensure patient involvement in decision making about choice of treatment and ensure support, regular follow-up, and tracking of all DMT-treated patients. This study was approved by the Calgary Research Ethics Board. All participants provided written informed consent. The supporting STROBE checklist is available as supporting information; see S1 STROBE Checklist.

### Study population

All adult (≥18 years old) patients with RRMS who started an injectable DMT between January 1^st^, 1996 and July 1^st^, 2011 at the Calgary MS Clinic were included in this study. All participants met the 2005 McDonald criteria for MS and had either two relapses, or one relapse and a chronologically (≥3 months) separate gadolinium enhancing lesion on MRI, within the immediate 2 year period preceding DMT start [12]. Those with clinically isolated syndrome (CIS) and most patients with progressive disease were not eligible for government-funding, and progressive MS at treatment initiation was uncommon at our clinic, therefore, these patients were excluded from this analysis. Those who previously participated in a clinical trial, initiated DMT before attending our clinic, began a second-line or oral therapy first, or did not provide written informed consent were also excluded. The sample size was determined by the number of patients who met the eligibility criteria.

### Outcomes

Persistence was defined as the time-to-discontinuation of all injectable therapies. The time-to-discontinuation of the first injectable DMT was also determined. Persistence was measured by calculating the number of days between treatment initiation and discontinuation. Ongoing use of treatment was based on participant history at their annual DMT review visit. A period without treatment of more than 90 days during treatment or between a switch was classified as discontinuation. Switching from one IFN-β formulation to another was considered continuous IFN-β treatment if the gap between drugs was not more than 90 days. Switching to a second-line or oral therapy (mitoxantrone, cyclophosphamide, natalizumab, azathioprine, fingolimod, or dimethyl fumarate) was also defined as injectable discontinuation. Participants were censored on the date they were last known to be taking DMT if they: (1) discontinued treatment due to pregnancy, (2) moved and were no longer followed in the Calgary MS Clinic, (3) were lost to follow-up, or (4) died. Those who were believed to still be on therapy as of February 1^st^, 2014 were censored on the date of their last clinic visit. Additionally, to evaluate the impact of the decision to limit the gap off treatment to 90 days we evaluated persistence using the definition of an off-treatment gap of 60 or 180 days [13,14].

### Statistical analysis

The three IFN-β formulations were combined into a single IFN-β group after we confirmed that there was no difference in persistence between the two subcutaneous formulations of IFN-β (S1 Table) and decided that the number of patients treated with intramuscular IFN-β was too low (34/565) for it to remain a separate treatment group. DMT group comparisons (GA and IFN-β) were made using chi-square tests, t-tests, or Wilcoxon rank-sum tests, as appropriate. Median time-to-discontinuation of all first-line injectable DMTs, and the first injectable DMT type used, and 95% confidence intervals (CIs) were calculated using the Kaplan-Meier survival method. Potential predictors of persistence were age at index, gender (women, men), EDSS score at index, time from MS disease onset to the index date, initial DMT prescribed (GA, IFN-β), and initiation period (1996–2000, 2001–2005, 2006–2011). Survival curves were compared between groups using log-rank tests. Clinically meaningful cut-points were used to categorize continuous and ordinal variables: age (<30, 30–39, 40–49, and ≥50 years), EDSS score (<2, 2–3.5, and ≥4), and MS duration (<2, 2–4, 5–9, and ≥10 years). Age was subsequently dichotomized as <30 years and ≥30 years because there were no differences between the older age groups (data not shown). We used separate multivariable Cox regression models to investigate the impact of baseline characteristics on persistence with all DMTs and the first DMT type used, and report hazard ratios and 95% CIs as measures of association. The proportional hazards assumption was evaluated using statistical tests based on the Schoenfeld residuals and plots of the Martingale residuals were used to assess the linearity assumption of continuous covariates. Goodness-of-fit of the regression models was tested using the Groennesby and Borgan test. We also report reasons for discontinuing the initial DMT and describe utilization of DMTs after patients discontinued all injectable DMT. Statistical significance was based on a two-sided *p* value of 0.05 and all analyses were performed with the statistical software package STATA, version 11.2 (StataCorp, 2009).

## Results

### Study cohort characteristics at the date of treatment initiation

**Fig 1:**
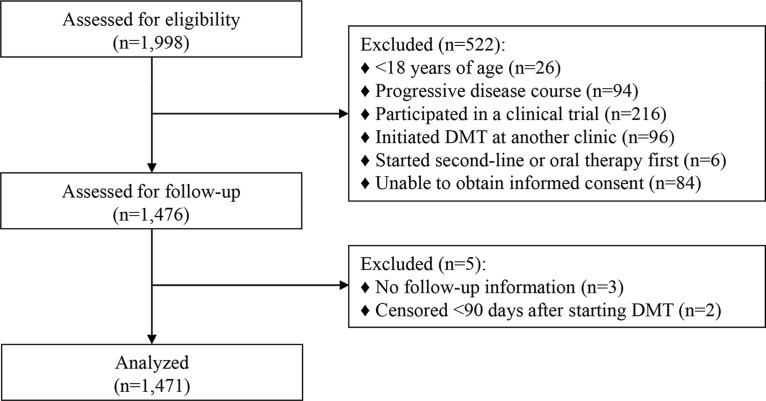
Flow diagram of patient selection into the study cohort.

Of 1998 patients who started a first-line DMT, 1476 met the study criteria (Fig 1). Of these, five were excluded because three had no follow-up information and two were censored less than 90 days after initiating DMT. Thus, the survival analyses included 1471 patients. Six participants were missing EDSS scores at treatment initiation so they were removed from multivariable analyses and the reason for stopping their first DMT was unknown for 9 patients; data was otherwise complete. The baseline characteristics of the study cohort are presented in Table 1.

**Table 1:** Characteristics of the study cohort at the index date overall and by the initial DMT prescribed.

Characteristic	Overall (n = 1,471)	GA (n = 906)	IFN-β ^a^ (n = 565)	*p* value
**Age (years)**, mean (SD)	38.4 (9.3)	38.6 (9.1)	38.0 (9.7)	0.28 ^c^
**Gender**, n (%)				0.09 ^d^
Women	1,108 (75.3)	696 (76.8)	412 (72.9)	
Men	363 (24.7)	210 (23.2)	153 (27.1)	
**EDSS** ^b^, median (IQR), range	2.0 (1.5–3.0), 0–8.0	2.0 (1.5–3.0), 0–8.0	2.0 (1.5–3.5), 0–7.5	0.002 ^e^
**MS Duration (y)**, mean (SD), range	6.0 (6.7), 0–48	6.2 (6.5), 0–38	5.8 (7.8), 0–48	0.34 ^c^
**Initiation Period**, n (%)				0.26 ^d^
1996–2000	440 (29.9)	281 (31.0)	159 (28.1)	
2001–2005	546 (37.1)	322 (35.5)	224 (39.6)	
2006–2011	485 (33.0)	303 (33.4)	182 (32.2)	

GA = glatiramer acetate; IFN-β = interferon-beta; EDSS = Expanded Disability Status Scale; y = years.

^a^IFN-β-1a s.c. (n = 411), IFN-β-1b (n = 120), IFN-β-1a i.m. (n = 34).

^b^Missing in 6 patients; of 8 patients with EDSS > 6.5 one had a comorbid condition that caused quadriparesis and seven were in the midst of a relapse.

^c^t-test.

^d^Chi-square test.

^e^Wilcoxon rank-sum test.

### Persistence with all injectable DMTs

**Fig 2:**
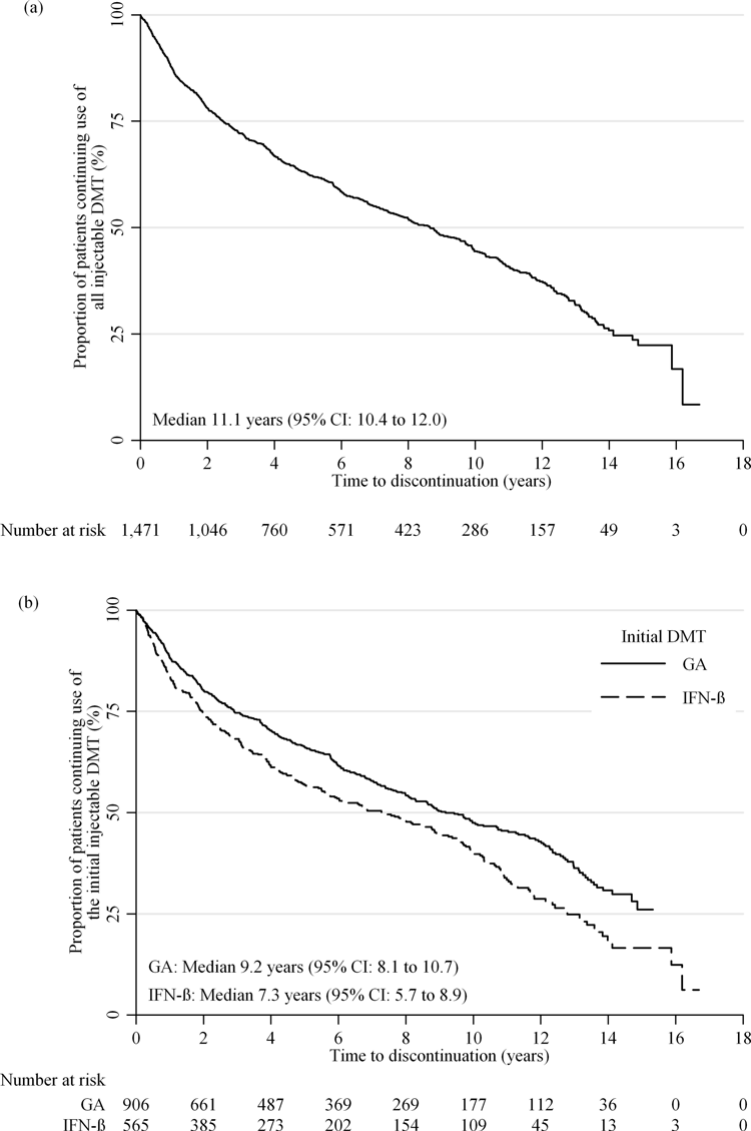
Time to discontinuation of all (a) and the initial (b) first-line injectable DMT.

The mean (SD) follow-up time for this cohort was 6.1 (4.4) years (range 0–18). From Kaplan-Meier survival analysis, the median (95% CI) time-to-discontinuation of injectable DMT was 11.1 (10.4–12.0) years (Fig 2a). When persistence was defined as an off-treatment gap of 60 or 180 days, the median time-to-discontinuation of all first-line DMTs was similar: 10.6 (9.9–11.1) and 11.6 (10.9–12.3) years, respectively. In multivariable Cox regression analysis the risk of discontinuing injectable treatment was greater for participants younger than 30 years at treatment initiation compared to all other age groups (Table 2). Those who initiated DMT between 2006 and 2011 had shorter persistence compared to all earlier time periods. Finally, the risk of discontinuing treatment increased with greater baseline EDSS score. Gender, MS duration and initial DMT prescribed were not associated with the risk of discontinuing injectable treatment.

**Table 2:** Kaplan-Meier and multivariable Cox regression analysis of potential factors associated with time-to-discontinuation of all first-line DMTs and the initial DMT prescribed.

		*All DMTs*				*Initial DMT*			
Covariate (at index)	n	Median Time to Discontinuation (y) (95% CI)	*p* value^a^	Hazard Ratio (95% CI)	*p* value^b^	Median Time to Discontinuation (y) (95% CI)	*p* value^a^	Hazard Ratio (95% CI)	*p* value^b^
***Overall***	1,471	11.1 (10.4–12.0)				8.6 (7.7–9.5)			
**Age (y)**			**<0.0001**		**<0.0001**		**<0.0001**		**<0.0001**
18–30	274	7.8 (6.0–9.9)		1.00		5.0 (3.7–6.3)		1.00	
30–73	1,197	11.8 (11.0–12.4)		0.61 (0.50–0.76)		9.4 (8.3–10.3)		0.63 (0.52–0.76)	
**Gender**			0.50		0.69		0.56		0.06
Women	1,108	11.3 (10.6–12.1)		1.00		8.3 (7.4–9.7)		1.00	
Men	363	10.6 (8.9–12.4)		0.96 (0.80–1.16)		9.1 (6.8–10.3)		0.85 (0.71–1.01)	
**EDSS**			**0.02**		**0.0006**		**0.003**		**0.0001**
0–1.5	475	12.3 (10.8–13.6)		1.00		8.9 (7.0–10.9)		1.00	
2–3.5	797	11.6 (10.3–12.3)	0.43	1.21 (1.00–1.46)	**0.05**	8.9 (7.9–10.0)	0.90	1.09 (0.92–1.30)	0.30
4–8	193	9.0 (6.9–10.8)	**0.004**	1.66 (1.28–2.14)	**0.0001**	5.6 (3.9–8.6)	**0.003**	1.64 (1.30–2.07)	**<0.0001**
**MS Duration (y)**	—	—	0.22	1.00^c^ (0.99–1.01)	0.96	—	0.08	1.00^c^ (0.98–1.01)	0.45
**Initial DMT**			**0.01**		0.07		**0.0001**		**0.008**
GA	906	12.1 (10.8–12.6)		1.00		9.2 (8.1–10.7)		1.00	
IFN-β	565	10.4 (9.5–11.1)		1.11 (1.00–1.23)		7.3 (5.7–8.9)		1.29 (1.11–1.50)	
**Initiation Period**			**<0.0001**		**<0.0001**		**<0.0001**		**<0.0001**
1996–2000	440	12.5 (11.4–13.3)		1.00		10.1 (8.9–11.3)		1.00	
2001–2005	546	11.8 (10.9–12.4)	0.58	1.10 (0.89–1.35)	0.38	10.3 (9.3–11.8)	0.75	0.99 (0.82–1.19)	0.89
2006–2011	485	6.0 (5.5–6.6)	**<0.0001**	2.45 (1.95–3.09)	**<0.0001**	4.6 (4.0–5.5)	**<0.0001**	2.24 (1.83–2.75)	**<0.0001**
GOF test				χ^2^, 9 = 5.09, *p* = 0.83				χ^2^, 9 = 11.60, *p* = 0.24	

DMT = disease modifying therapy; EDSS = Expanded Disability Status Scale; GA = glatiramer acetate; IFN-β = interferon-beta; y = years, GOF = goodness-of-fit; n = 1465 in multivariable models.

^a^Log-rank test.

^b^Wald test from multivariable Cox regression model adjusted for all covariates.

^c^Per one-unit increase in MS duration.

### Persistence with the first injectable DMT

The mean (SD) follow-up time for this cohort was 5.5 (4.3) years (range 0–17). The median (95% CI) time to stopping or switching the first injectable DMT was 8.6 (7.7–9.5) years. The results were similar when a 60 or 180 day gap was used to define persistence: 8.6 (7.7–9.5) and 8.8 (8.0–9.8) years, respectively. In the adjusted Cox regression analysis, time-to-discontinuation was again associated with age, EDSS, and period of treatment initiation. In addition, participants initially prescribed IFN-β had a greater risk of stopping or switching treatment than patients initially prescribed GA (Fig 2b). The stop/switch rate was 46% for GA and 56% for IFN-β over the entire study duration. Stop/switch rates over time for GA and IFN-β respectively were: 1 year (12% and 16%), 3 years (25% and 32%), 5 years (34% and 43%), 10 years (53% and 60%), and 12 years (57% and 71%). Gender and MS duration were not associated with the risk of stopping/switching the first injectable DMT used.

### Reasons for stopping injectable DMT

The first DMT was stopped by 733 (50%) patients; 46% (417/906) of those who started GA and 56% (316/565) of those who started IFN-β. The primary reasons for stopping the first DMT were intolerance (n = 352, 48%), inefficacy (n = 246, 34%), patient choice (n = 57, 8%), cost (n = 25, 3%), injection issues (n = 18, 3%), high levels of neutralizing antibodies (n = 18, 3%), unknown (n = 9, 1%), and unrelated medical problems or personal issues (n = 8, 1%). The proportion of participants who discontinued treatment due to intolerance was somewhat higher for IFN-β (152/565, 27%) compared to GA (200/906, 22%) (p = 0.04). Discontinuation due to inefficacy did not differ between the GA (155/906, 17%) and IFN-β (91/565, 16%) groups (p = 0.62).

There were 738 participants censored in the analysis of persistence with the first DMT used. The most common reason for censoring was reaching the end of follow-up (61%). This was followed by moving away (20%), pregnancy (13%), being lost to follow-up (4%), and death (2%). The data were similar for analysis of persistence with all DMTs: 610 patients stopped treatment and 861 were censored (data not shown).

### Use of DMT after stopping injectable therapy

Of the 1471 participants included in the survival analysis, 610 discontinued all injectable therapy for at least 90 days. Fifty-four percent (331/610) however resumed DMT and 46% (279/610) remained off treatment. Of the 331 that resumed treatment, 47 (14%) started a second line therapy, 110 (33%) started oral therapy, and 174 (53%) restarted injectable therapy after a break of more than 90 days. Of the 331 who resumed, 60% began within 6 months; 81% within 12 months.

## Discussion

In this long-term, population-based cohort study, we examined persistence with all injectable DMTs and with the first prescribed injectable DMT (GA or IFN-βs) in people with RRMS. The median time-to-discontinuation of all injectable DMTs was 11.1 years and the median time-to-discontinuation of the first prescribed injectable DMT was 8.6 years. Stop/switch rates were lower for GA than IFN-β s at all time points. We found that participants with greater disability at treatment initiation, those who started treatment before age 30, and those who started treatment between 2006 and 2011 stopped injectable therapy sooner. Gender and MS duration did not impact persistence but those who were initially prescribed GA remained on their first treatment significantly longer than those who started with IFN-β. Also, after discontinuing injectable DMT, 54% (331/610) of participants started oral or second line therapy or later restarted an injectable.

The observation that participants younger than 30 years at treatment initiation remain on treatment for less time than older participants is consistent with previous research [13–18]. The reasons are unclear but treatment failure is easier to detect in younger patients because relapses and MRI inflammatory activity are more common [19]. This may result in a higher rate of changing to another DMT. We did not find that MS disease duration affected persistence so it is unlikely that having less time to accommodate to an MS diagnosis was a major factor.

Participants with greater disability at initiation were also less persistent. This has been reported by others [15–18]. It may be due to reduced tolerance or an increased rate of treatment failure. Identifying progressive disease is easier in more disabled patients and progression may not be impacted by treatment and may lead to earlier discontinuation. Reduced persistence in those with greater disability also supports the belief that DMTs are more effective when started earlier in the evolution of MS [20].

We did not find any gender difference in persistence but the impact of gender has been inconsistent across other studies [14,16,18,21,22]. This variability in the impact of gender on persistence suggests that either it is not a major factor or that cultural differences related to gender, affecting the patient or the care team, may play a role in some settings.

We found that participants who started treatment between 2006 and 2011 were less persistent compared to those who started treatment during earlier periods: 1996 to 2000 or 2001 to 2005. Two previous studies have also noted that those who started DMT recently were more likely to discontinue therapy [13,16]. In contrast, increased medication persistence has been reported in recent years in other conditions such as HIV/AIDS and cardiovascular disease [23,24]. There may be several reasons for this recent reduction in persistence in MS. Evidence has accumulated to guide use of MRI to monitor DMT effectiveness and testing for neutralizing antibodies to IFN-β has become more available; these both facilitate earlier prediction of breakthrough disease and may trigger a switch of therapy. Newly available treatment options such as oral therapies and greater treatment expectations also increase the chance of switching therapy. Furthermore, patients and physicians have become less tolerant of the side-effect burden associated with injectable DMTs with new options available. Finally, it is possible that our findings reflect fading patient and/or physician confidence in the benefits of injectable DMTs compared with newer therapies or non-pharmacological treatments. Our ongoing study of persistence will evaluate persistence with any DMT, not just injectable therapy.

We found that participants initially treated with GA persisted longer on their first treatment, compared to those initially treated with IFN-β. While most previous studies revealed similar persistence with GA and IFN-β [13,14,16,25] differences similar and opposite to our findings have also been observed [11,15,26,27]. The reason for this variability in persistence between studies is unclear. In our study it could be due, in part, to the greater baseline EDSS in participants starting IFN-β although this was accounted for in the multivariate model. It is true also that since treatment was not randomized other unrecognized differences between groups may have existed.

The main reason for discontinuation in this study was intolerance, followed by inefficacy. Significantly more patients treated with IFN-β than GA discontinued due to intolerance but there was no difference between the IFN-β and GA groups in discontinuation due to inefficacy. Previous studies of DMT persistence found that patients on IFN-β discontinued treatment predominantly due to intolerance [10,15,28], inefficacy [18,22,29], or both equally [17]. Only one other study compared reasons for discontinuation between IFN-β and GA [15]. Similar to our data, their results revealed that the most common reason for treatment discontinuation was intolerance, followed by inefficacy; however, the rate of discontinuation did not differ by reason, between treatments [15]. Variation in patient characteristics may account for the disparity between studies. However, these data do not provide a clear understanding of why patents discontinue treatment. There are likely to be several factors that influence discontinuation in many patients.

Median time-to-discontinuation in the Calgary cohort (8.7 years for first DMT and 11.1 years for all injectable DMTs) is much longer than in the majority of reported studies. For instance, among 760 RRMS patients from seven Australian MS clinics the median time-to-discontinuation was only 1.7 years for GA and 2.5 to 2.8 years for IFN-β [15]. Additionally, among 1896 MS patients from four MS clinics in British Columbia, Canada the median time-to-discontinuation was only 2.9 years for the initial injectable DMT type and 6.3 years for all injectable DMTs [16]. Twenty-five percent of the participants in their initial injectable DMT cohort interrupted therapy within 1 year. Even lower levels of persistence were found in an analysis of medication claims from Ontario, Canada (n = 682) where stop/switch rates ranged from 53 to 59% for GA/IFN-β after only 2 years [25].

A few studies reported similar or better levels of persistence than those found in our study; however, all of these were much smaller and shorter and did not include follow-up within the recent era of greater DMT choice [10,18,29]. The persistence rates for GA and IFN-β reported here are also substantially higher than for injectable treatments for Type-II diabetes, schizophrenia, and hepatitis C [30–38]. Therefore, in comparison with most studies of injectable therapies in MS and other chronic diseases, patients with MS in our clinic have been outstanding in their ability to remain on injectable therapy.

A variety of factors may explain the overall variability in persistence between studies. Differences known to impact persistence include education, adverse effects, increased disability, depression at treatment initiation, patient and physician treatment expectations, cost to patients, patient involvement in treatment selection, and the nature of the patient support and follow-up care. In our MS Clinic, we systematically educate and support patients from the first discussions regarding DMT and throughout their care and most choose their therapy with assistance from their physician and nurse. In addition, treatment cost was minimal to patients and did not differ between therapies. Other authors also cite quality of follow-up as a reason for high persistence rates in their MS clinic [18,29].

Study design likely also impacts results. Studies using aggregate data from claims or drug-plan databases or from multiple clinics have tended to report lower rates of persistence; this may reflect study of populations with variable patient education and support. They may also underestimate persistence as patients may continue therapy but change clinics or insurance plans. Likewise, studies that included SPMS patients may find shorter persistence because SPMS patients are more likely to be DMT non-responders. Finally, persistence is affected by how stopping due to pregnancy is managed in the study design. We chose to censor patients when they stopped treatment due to pregnancy because DMT is not indicated during pregnancy, discontinuation is usually temporary, and it did not reflect lack of effectiveness, tolerance, or willingness to continue treatment.

Strengths of this study include that data was prospectively collected, the large sample size (n = 1471), the long study duration (up to 18 years of injectable treatment) and the completeness of the data. There was complete persistence data in 87% of participants; only 29 (2%) were lost to follow-up and 160 (11%) moved away from Southern Alberta while still using DMT. To our knowledge, this study provides the longest period of follow-up of all persistence studies published to date and it is the largest from a single center. Also, unlike most studies, only patients who were DMT-naïve at treatment initiation and started their DMT at our clinic were included, thus assuring prospective and complete capture of data and standardization of patient support and management. It also assures that the data relate to an easily described sector of MS patients—adults with active RRMS who have not previously been treated with a DMT and are not involved in a clinical trial. Also, based on provincial administrative data, the study represents a community population with MS as 99% of people in Southern Alberta with MS who get neurologic care, get this care from the Calgary MS Clinic. Furthermore, only 5% of the eligible study cohort (84 patients) was excluded because they did not provide consent or because follow-up was not complete, only 2% of patients were lost to follow-up, and 11% moved away. We believe these findings can be generalized to a real-world setting where funding of treatment is continuously available and patients are well supported.

Importantly, we found that only 279 patients in our study (19% of the entire cohort of 1471 RRMS patients) discontinued injectable treatment without restarting later or switching to an oral or second-line therapy. Our data therefore shows that most patients remain treated. This places a very optimistic perspective on the value of treatment to patients with MS. Such a high rate of continued treatment also suggests that outcomes in this cohort will reflect the impact of treatment; analysis of treatment outcomes is ongoing. Longer follow-up of patients still on injectable therapy and further study of the patients who stopped DMT without resuming or starting a new therapy, those who resumed treatment or started a second-line or oral therapy, and those who stopped due to pregnancy will help us understand treatment patterns in RRMS. As oral therapies and additional second line therapies for MS become increasingly available treatment patterns are likely to evolve.

Overall, persistence in this study was high compared to similar studies. While some clinicians suggest that frequent physician follow-up of DMT-treated patients improves persistence, we provide evidence that a program with less frequent follow-up but with attention to issues that promote adherence, including education and support from expert nurses, also leads to excellent adherence. Evidence of excellent persistence will facilitate interpretation of treatment outcomes in this population.

## Supporting Information

S1 STROBE Checklist STROBE Statement. (DOC)

S1 Table Kaplan-Meier and Cox regression analysis comparing time-to-discontinuation of all and the initial first-line injectable DMT between subcutaneous formulations of IFN-β. (DOC)
