# Supplementary material for: Long-Term Persistence with Injectable Therapy in Relapsing-Remitting Multiple Sclerosis: An 18-Year Observational Cohort Study
Source: PLoS One. 2015 Apr 13;10(4):e0123824. doi: 10.1371/journal.pone.0123824 (PMC4395027; doi:10.1371/journal.pone.0123824)
Supplement: S1 Table — (DOC) [file pone.0123824.s002.doc]

**S1 Table.** Kaplan-Meier and Cox regression analysis comparing time-to-discontinuation of all and the initial injectable DMT between subcutaneous formulations of IFN-β.

|  |  | ***All DMTs*** | | | | ***Initial DMT*** | | | |
| --- | --- | --- | --- | --- | --- | --- | --- | --- | --- |
| **Initial DMT** | **n** | **Median Time to Discontinuation (y) (95% CI)** | **p value*** | **Hazard Ratio (95% CI)** | **p value†** | **Median Time to Discontinuation (y) (95% CI)** | **p value*** | **Hazard Ratio (95% CI)** | **p value†** |
| IFN-β-1a s.c. | 411 | 10.8  (9.6-11.1) | 0.82 | 1.00 | 0.82 | 7.7  (5.8-9.6) | 0.46 | 1.00 | 0.46 |
| IFN-β-1b | 120 | 9.9  (6.8-15.9) |  | 0.96  (0.71-1.31) |  | 8.0  (4.8-10.6) |  | 0.90  (0.68-1.19) |  |

* Log-rank test; † Wald test from Cox regression model; IFN-β = interferon-beta.
